# Distal radius fracture – Supination underestimates dorsal tilt in distal radius fracture radiographs: a case report

**DOI:** 10.1007/s10140-025-02367-w

**Published:** 2025-07-09

**Authors:** Sebastian P. L. Clark, Maria Ilsø, Jens C. Werlinrud, Benjamin S. Rasmussen, Janni Jensen

**Affiliations:** 1 Research and Innovation Unit of Radiology, University of Southern Denmark, Kløvervænget 10, 2, Odense, 5230 Denmark; 2 Department of Radiology, Odense University Hospital, Odense, 5000 Denmark; 3 Department of Orthopaedic Surgery, Odense University Hospital, Odense, 5000 Denmark

**Keywords:** Distal radius fracture, Dorsal Tilt, Dorsal angulation, Radiographic measurement, Scaphopisocapitate alignment

## Abstract

Distal radius fractures are one of the most prevalent fracture types. Dorsal tilt of the articular surface of the distal radius as assessed on the lateral radiograph is often used in determining the correct treatment method. This report seeks to highlight the importance of correct forearm positioning when obtaining distal radius radiographs, and the effects of pronation and supination on dorsal tilt measurements of the radius. It presents a case of a 35-year-old male with a malaligned left-sided distal radius fracture sustained during a sports-related fall.

**Supplementary Information:**

The online version contains supplementary material available at 10.1007/s10140-025-02367-w.

## Graphical Abstract

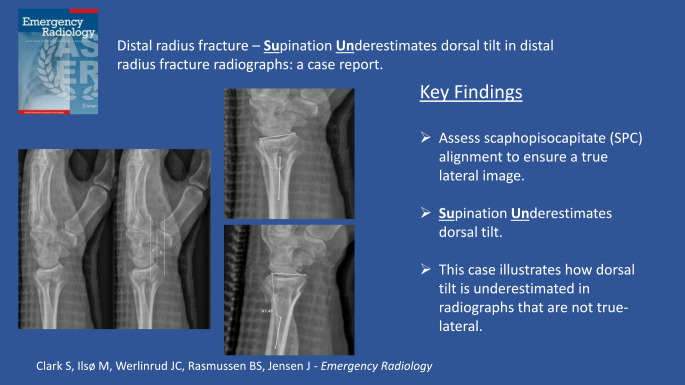

## Introduction

**Fig. 1:**
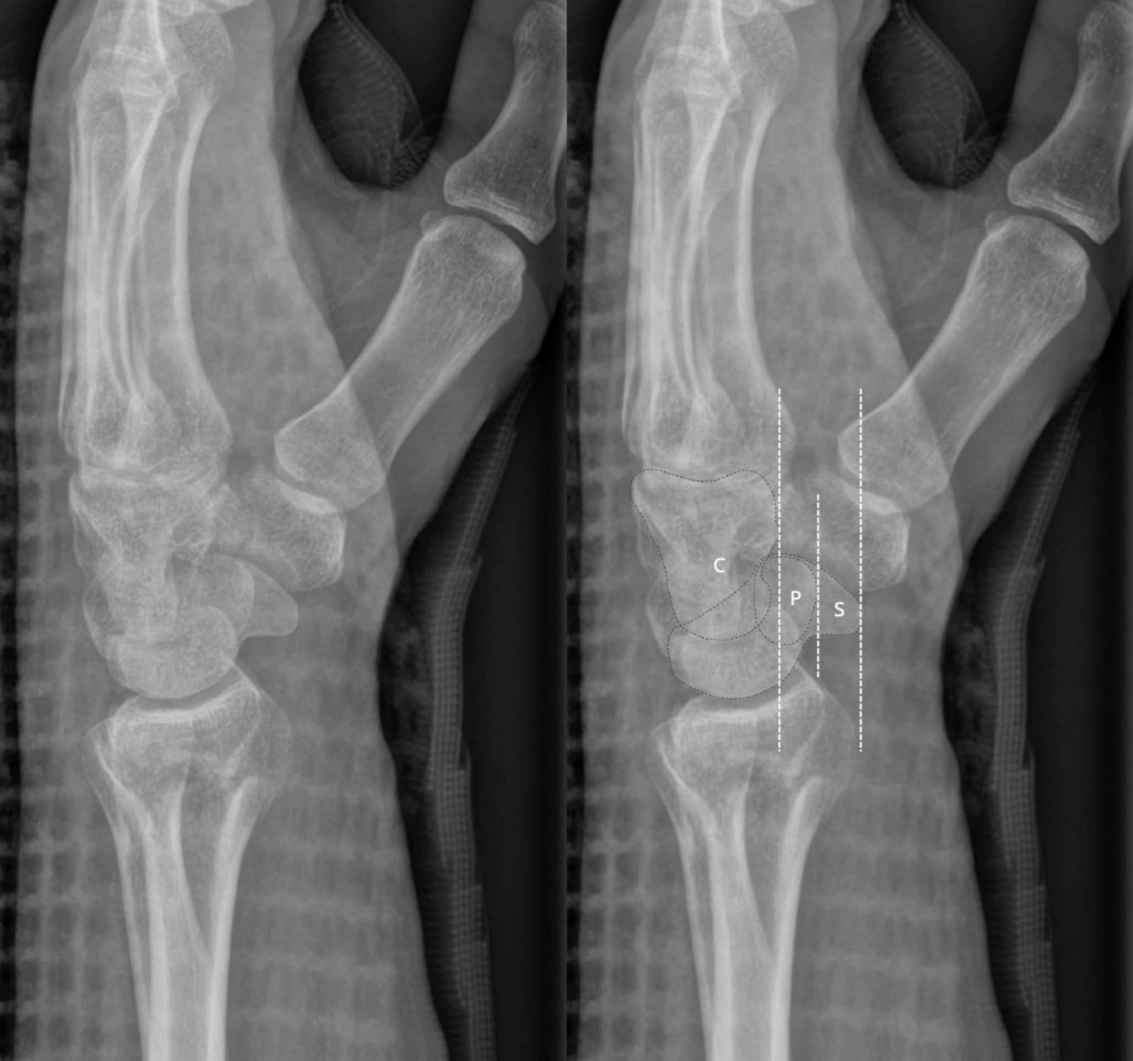
A true lateral radiograph illustrating the scaphopisocapitate alignment where the palmar cortex of the pisiform is positioned over the central third of the interval between the palmar cortices of the distal scaphoid pole and the capitate head

Distal radius fractures (DRFs) are one of the most prevalent fracture types that occur in the adult population. The American Academy of Orthopaedic Surgery (AAOS) and the Danish National Guidelines on Distal Radius Fractures both agree that post-reduction DRFs with a radiographic dorsal tilt of at least 10° or greater from the neutral axis of the radius could require open reduction and internal fixation. However, the age of the patient and activities of daily living, are likewise factors used in determining the appropriate treatment method [1, 2]. Hence, the radiographic measurement of dorsal tilt plays a key role in guiding the treatment decisions for DRFs. Kreder et al. proposed standards for measuring dorsal tilt as an angle between the central longitudinal axis of the distal radius and a line connecting the dorsal and volar margins of the distal articular surface of the radius [3]. Several authors have highlighted the importance of correct forearm positioning when acquiring a true lateral wrist radiograph and the subsequent measurement of dorsal tilt, as well as the inter- and intraobserver dependencies while measuring true lateral, supinated and pronated wrists [3–7]. However, the recurrent issue in standardizing measurements of dorsal tilt is that different methods of defining a true lateral wrist radiograph have been suggested. Yang et al. proposed the scaphopisocapitate (SPC) alignment to radiographically assess the true lateral position as opposed to distal radioulnar overlap [8]. An example of the SPC alignment on a true lateral radiograph is illustrated in Fig. 1. Jensen et al. and Pennock et al. further investigated the effects of pronation and supination on dorsal tilt measurements using radiostereometric analysis on both fractured and non-fractured specimens and concluded that supination during acquisition of the lateral wrist radiograph underestimates the measured value of dorsal tilt by up to 0.3–0.68° for every degree of supination. The opposite would be true for pronation [4, 6, 7].

Post-traumatic arthritis (PA) is a recognized complication following distal radius fractures. The condition often manifests as joint pain, stiffness and reduced functionality over time. A systematic review found a high prevalence of the development of PA following a distal radius fracture in non-osteoporotic patients and that articular incongruence was a significant predictor for PA. Other radiographic measurements such as radial length, radial inclination, dorsal angulation and ulnar variance were presented in literature as predicting factors for PA with conflicting results. It is emphasized that these conflicting results could be due to a substantial variability in how these measurements are defined in literature which complicates their predictive value and underscores the need for standardized guidelines to ensure consistency when interpreting different radiographic measurements [9].

### Case report

A 35-year-old male presented in the emergency department with a malaligned left wrist following a high-energy trauma during sports. The initial objective clinical examination noted a distal radius fracture with malalignment, bruising and discoloring of the wrist. The patient had normal neurovascular function. In accordance with normal procedure, the patient underwent routine clinical and radiological follow-ups and approximately two weeks after initial injury it was decided to perform surgery. At that specific follow-up, two lateral radiographs of the left wrist were taken sequentially within a time frame of one minute, both of which indicated the extra-articular oblique distal radius fracture diagnosed at the time of the injury. However, the interesting aspect in the two lateral radiographs obtained less than one minute apart was, that the measured value of the dorsal tilt changed significantly over the course of one minute or at least mimicked a change. The measured angulation of the distal radius changed from 2.5° of dorsal tilt in the first lateral radiograph to 14° of dorsal tilt in the subsequent lateral radiograph, which was acquired one minute later. At closer inspection of the two lateral radiographs, one of the radiographs was rotated. The establishment of the true lateral image was done by assessing the SPC alignment, as proposed by Yang et al. [8]. The measurements of dorsal tilt were conducted by an expert diagnostic radiographer and a radiology resident using the method proposed by Kreder et al. [3].

**Fig. 2:**
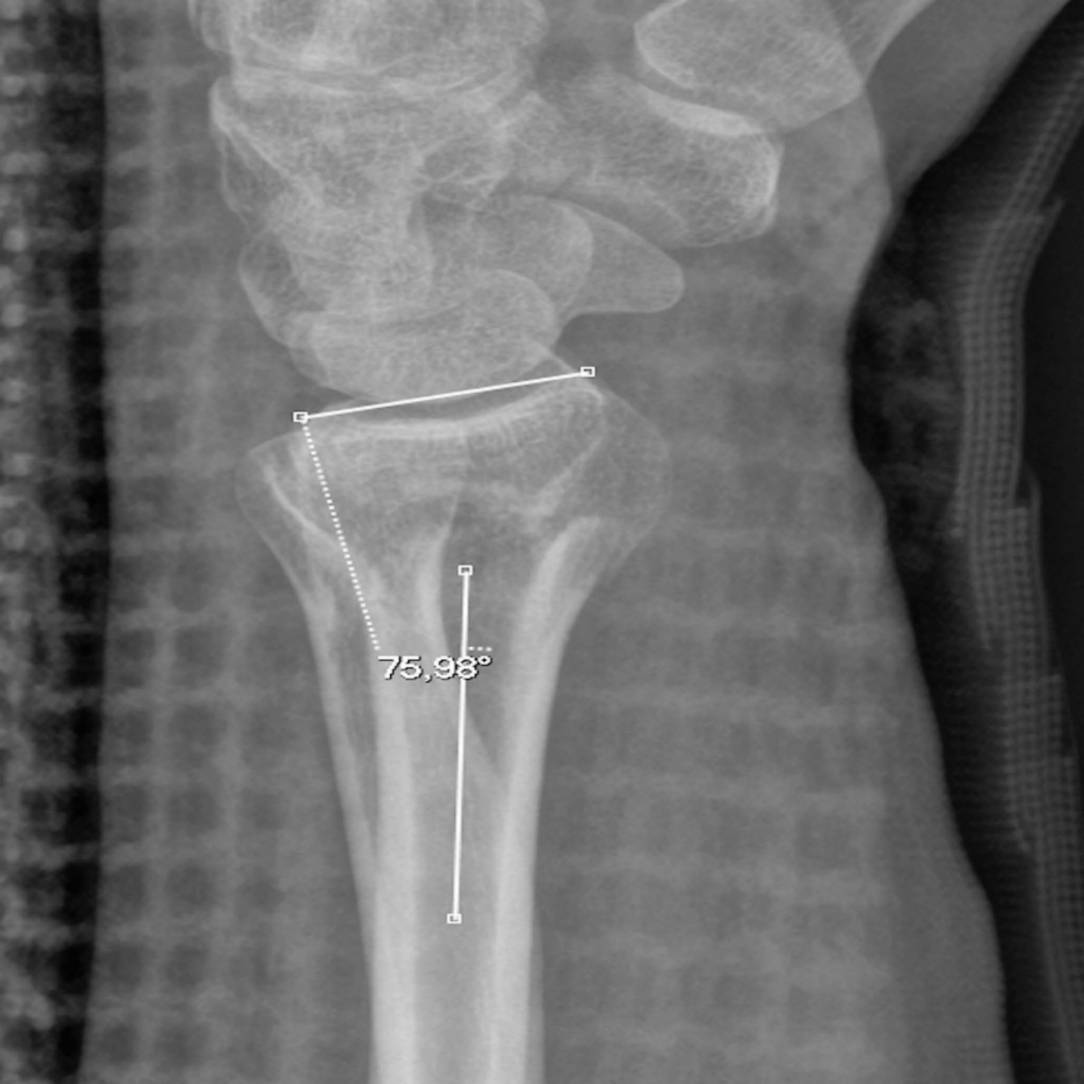
**35**-year-old male with left sided extraarticular distal radius fracture. Figure 2 shows the true lateral radiograph. Notice the scaphopisocapitate criterium and distal radioulnar overlap, as well as measured dorsal tilt

**Fig. 3:**
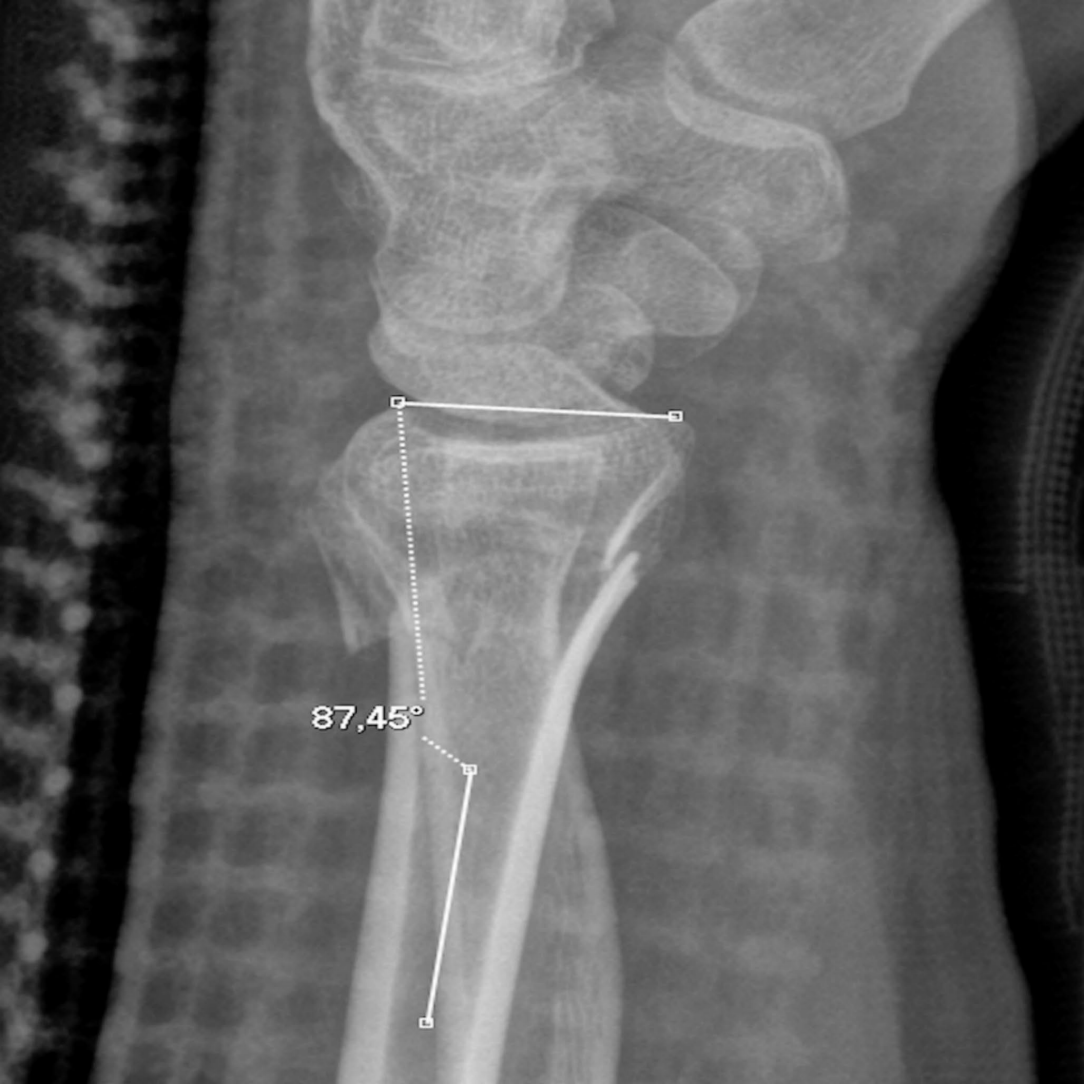
**35**-year-old male with left sided extraarticular distal radius fracture. Figure 3 shows the supinated lateral radiograph. Notice the scaphopisocapitate criterium and distal radioulnar overlap, as well as measured dorsal tilt

Figure 2 shows the true lateral radiograph, as the ***volar*** surface of the pisiform is positioned in the central third of an interval defined by the distance between the volar cortices of the capitate and the scaphoid bone. Figure 3 shows the supinated lateral radiograph with the ***dorsal*** cortex of the pisiform bone intersecting the central third of the volar aspect of the scaphoid bone, and the volar cortex of the pisiform bone protruding anteriorly to the tubercle of the scaphoid bone. The volar or dorsal position of the pisiform bone can thus be used to assess pronation and supination of the wrist itself. Figure 3 would be assessed as an overly supinated radiograph, as the pisiform bone’s volar surface is volarly positioned compared to the tubercle of the scaphoid bone.

Figure 3 also illustrates how using true distal radioulnar overlap could lead to change in apparent measurements, particularly in the case of a dislocated fracture. The illustrated measurements show a difference of up to 11.5° (from 2.5° dorsal angulation to 14° of dorsal angulation) on respectively the supinated and the true-lateral images, thus illustrating how **Su**pination **Un**derestimates Dorsal Tilt. This also illustrates the possible consequence that mal-positioned lateral radiographs of the distal radius could have for the patient’s treatment method, as the first supinated radiograph would potentially not have led to open reduction and internal fixation, as opposed to the true lateral image acquired one minute later. While dorsal angulation is an important consideration, other factors such as intra-articular involvement, fracture displacement, and patient-specific factors (e.g., functional demands, comorbidities) similarly play a role in treatment decisions. In this case, however, surgery was ultimately performed in accordance with Danish national guidelines.

## Discussion

The overall objective of this report was to highlight the importance of correct forearm positioning when obtaining lateral radiographs of the wrist. Our case illustrates the importance of obtaining a true lateral radiograph when assessing dorsal tilt, which may be important when determining treatment method and is equally important in follow-up imaging where radiographs obtained with opposing forearm rotation may mimic fracture instability.

We illustrated the need for a consensus on true lateral radiographs using reliable anatomical structures as proposed by Yang et al. instead of other typically used methods such as distal radioulnar overlap [8]. Moreover, several studies have reported that inter-observer agreement in measuring dorsal tilt is generally moderate and can be further affected by factors such as forearm positioning - particularly the degree of supination or pronation from the neutral axis - as well as the experience level of the observers [3, 4, 6, 7]. We also suggest consensus in the way of obtaining measurements systematically, such as proposed by Kreder et al. [3]. This case could potentially benefit all readers regardless of the experience level of the reader, and since dorsal tilt is used in some national guidelines [1, 2, 10] it could help in distinguishing between fractures requiring surgical intervention or conservative treatment.

One might speculate that there are some limitations in this field of study such as limited research on quantitative measurements of distal radius fractures with intraarticular involvement, simultaneous carpal instability, ligament injury, or in patients with severe osteoarthritis or rheumatological disease. This could potentially affect the mean measurement errors – and thus lead to inaccurate patient-reported outcomes.

This report underlines the importance of accurate and standardized radiographic evaluation of distal radius fractures, particularly when assessing dorsal tilt. Variability in forearm positioning such as supination or pronation, can further complicate the interpretation of radiological parameters, potentially leading to misdiagnosis or suboptimal management. By ensuring standardized imaging and consistent interpretation, the risk of long-term complications may potentially be minimized, ultimately improving patient outcomes and guiding appropriate treatment decisions.

When in doubt, assess the scaphopisocapitate alignment and remember that **Su**pination **Un**derestimates Dorsal Tilt.

## Electronic supplementary material

Below is the link to the electronic supplementary material.

Supplementary Material 1

## Data Availability

The images used in this study have not been manipulated and are available to the Journal of Emergency Radiology at request.
