# Supplementary material for: Distal radius fracture – Supination underestimates dorsal tilt in distal radius fracture radiographs: a case report
Source: Emerg Radiol. 2025 Jul 9;32(4):663–8. doi: 10.1007/s10140-025-02367-w (PMC12328503; doi:10.1007/s10140-025-02367-w)
Supplement: Supplementary file 1 — Supplementary Material 1 [file 10140_2025_2367_MOESM1_ESM.pptx]

## Slide 1
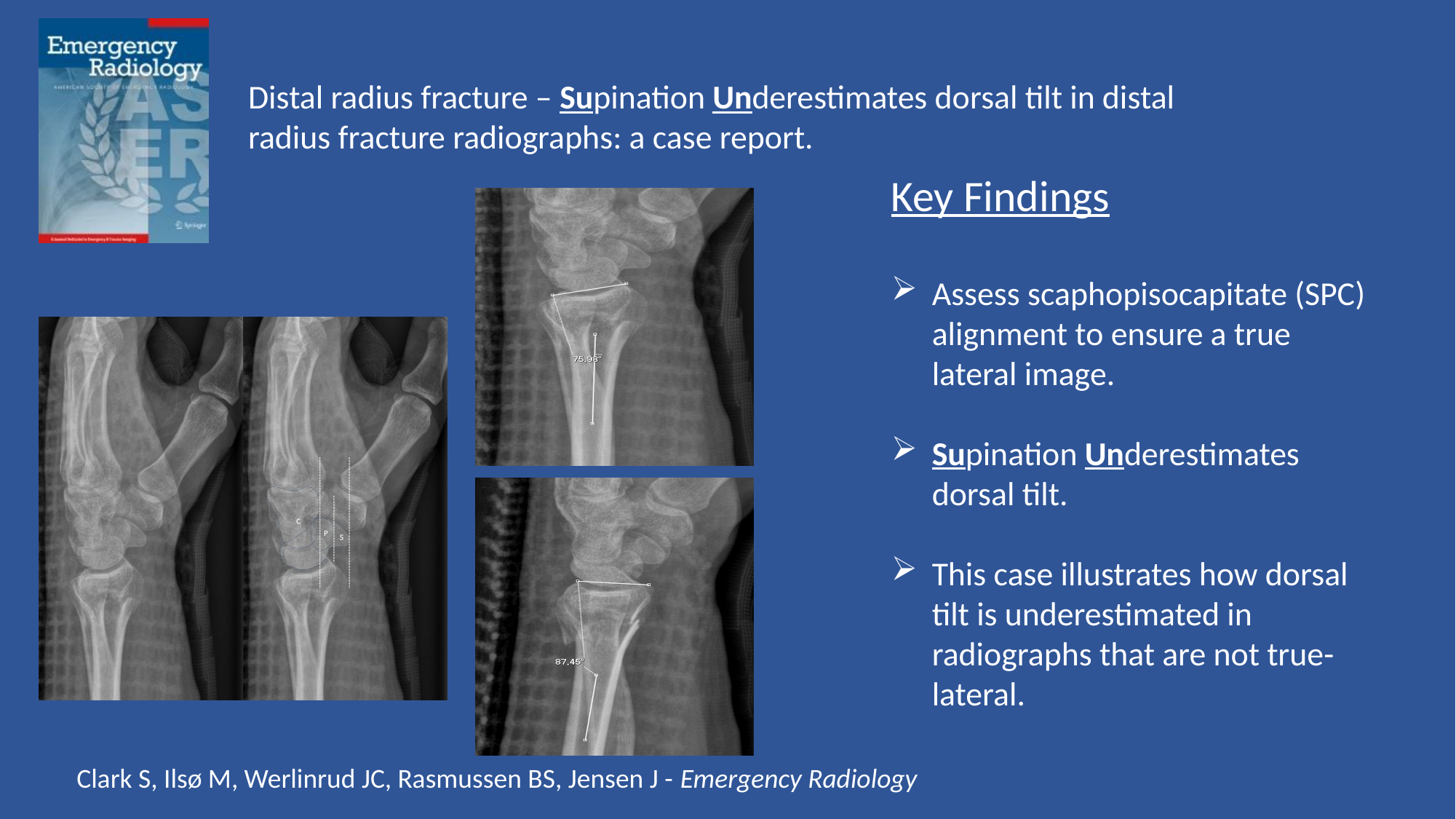

Distal radius fracture – Supination Underestimates dorsal tilt in distal radius fracture radiographs: a case report.
Key Findings
Assess scaphopisocapitate (SPC) alignment to ensure a true lateral image.
Supination Underestimates dorsal tilt.
This case illustrates how dorsal tilt is underestimated in radiographs that are not true-lateral.
Clark S, Ilsø M, Werlinrud JC, Rasmussen BS, Jensen J - Emergency Radiology
